# Assessing the Policy Environment for Active Mobility in Cities—Development and Feasibility of the PASTA Cycling and Walking Policy Environment Score

**DOI:** 10.3390/ijerph18030986

**Published:** 2021-01-22

**Authors:** Sonja Kahlmeier, Esther Anaya Boig, Alberto Castro, Emilia Smeds, Fabrizio Benvenuti, Ulf Eriksson, Francesco Iacorossi, Mark J. Nieuwenhuijsen, Luc Int Panis, David Rojas-Rueda, Sandra Wegener, Audrey de Nazelle

**Affiliations:** 1 Department of Health, Swiss Distance University of Applied Science (FFHS), 3900 Brig, Switzerland; 2 Biostatistics and Prevention Institute (EBPI), Epidemiology, University of Zurich, 8001 Zurich, Switzerland; alberto.castrofernandez@swisstph.ch; 3 Centre for Environmental Policy, Imperial College London, London SW7 1NE, UK; e.anaya-boig14@imperial.ac.uk (E.A.B.); anazelle@imperial.ac.uk (A.d.N.); 4 Technology, Engineering and Public Policy (STEaPP), Department for Science, University College London, London WC1E 6JA, UK; emilia.smeds@ucl.ac.uk; 5 Roma Servizi per la mobilità, 00143 Rome, Italy; fabrizio.benvenuti@agenziamobilita.roma.it (F.B.); francesco.iacorossi@agenziamobilita.roma.it (F.I.); 6 Trivector, 11123 Stockholm, Sweden; ulf.eriksson@sll.se; 7 Institute for Global Health (IS Global), 08003 Barcelona, Spain; mark.nieuwenhuijsen@isglobal.org; 8 Flemish Institute for Technological Research VITO, 2400 Mol, Belgium; luc.intpanis@vito.be; 9 School for Mobility, Hasselt University, 3500 Hasselt, Belgium; 10 Barcelona Institute for Global Health (ISGlobal), 08003 Barcelona, Spain; David.Rojas@colostate.edu; 11 Environmental and Radiological Health Sciences, Colorado State University, Fort Collins, CO 80523-1601, USA; 12 Institute for Transport Studies (ITS), University of Natural Resources and Life Sciences BOKU, 1180 Vienna, Austria; sandra.wegener@boku.ac.at

**Keywords:** cycling, walking, active travel policy assessment, scoring, transport planning

## Abstract

The importance of setting a policy focus on promoting cycling and walking as sustainable and healthy modes of transport is increasingly recognized. However, to date a science-driven scoring system to assess the policy environment for cycling and walking is lacking. In this study, spreadsheet-based scoring systems for cycling and walking were developed, including six dimensions (cycling/walking culture, social acceptance, perception of traffic safety, advocacy, politics and urban planning). Feasibility was tested using qualitative data from pre-specified sections of semi-standardized interview and workshop reports from a European research project in seven cities, assessed independently by two experts. Disagreements were resolved by discussions of no more than 75 minutes per city. On the dimension “perception of traffic safety”, quantitative panel data were used. While the interrater agreement was fair, feasibility was confirmed in general. Validity testing against social norms towards active travel, modal split and network length was encouraging for the policy area of cycling. Rating the policy friendliness for cycling and walking separately was found to be appropriate, as different cities received the highest scores for each. Replicating this approach in a more standardized way would pave the way towards a transparent, evidence-based system for benchmarking policy approaches of cities towards cycling and walking.

## 1. Introduction

Insufficient physical activity (PA) is a key cause of non-communicable diseases, including type 2 diabetes, stroke, cardiovascular diseases, cancers, and poor mental health, as well as premature mortality [1]. In 2016, the prevalence of insufficient physical activity was about 28% for adults globally, and evidence of any improvements in its prevalence were scarce [2,3]. This challenge is unlikely to be solved by classical health promotion approaches alone, such as organized forms of sport or leisure time exercise. Cycling and walking have been increasingly recognized as promising approaches to promote regular physical activity among wide ranging population groups [4,5,6,7]. The WHO European Strategy on Physical Activity launched in 2016, includes a specific objective to reduce car traffic and to increase walking and cycling [8]. Promoting physical activity through cycling and walking as a means of travel also contributes to sustainable urban environments [9,10,11] and to addressing climate change [12,13,14].

It has been underlined that the policy environment has a decisive influence on promoting physical activity, including cycling and walking [2,3,4,10,11]. For example, whether walking or cycling are culturally well-regarded or whether there are influential advocacy groups that promote active travel, may influence the likelihood of active travel policies being properly and effectively implemented. The effectiveness of a new cycle lane in increasing cycling may be greater in an area where cycle-aware planning departments are likely to be aware of the best practice for such types of infrastructure and implement a bundle of cycle-promoting policies in conjunction. Thus, insight into the policy and cultural context is important to identify good practices and to derive lessons for policy makers. There are few tools available, however, to assess this policy context. An audit tool has been developed to qualitatively assess national approaches to physical activity promotion [15]. Quantitative scoring systems exist to audit local physical environments for cycling and walking [16,17,18]. There is also a bicycle policy audit based on a guided self-assessment [19]. To date, however, no scientifically based scoring systems have been developed to specifically assess the local policy and social environment for cycling and walking. This hampers comparability of approaches across cities and countries and the identification of success factors and barriers to walking and cycling promotion. In addition, a score capturing this vitally important determinant could also be included into multivariate models to better understand the interplay of determinants of cycling and walking behavior.

This paper describes the development of a science-embedded, evidence-based approach to scoring the friendliness of the policy environment for walking and cycling promotion in cities, including the social, policy and planning context, and it presents the results of a feasibility study in seven case study cities across Europe. We will first explain the methodological approach taken and data used, followed by presenting the resulting scoring system for assessing the policy environment for walking and for cycling and its application to the Physical Activity through Sustainable Transport Approaches (PASTA) case study cities (CSC). Thereafter, the results of an extensive validity testing analysis will be summarized and discussed.

## 2. Materials and Methods

### 2.1. PASTA Project

This study was part of the “Physical Activity through Sustainable Transport Approaches” (PASTA) project. PASTA is a mixed-method and multilevel design research project on active mobility (AM) which spans disciplines, research and practice, determinants and impacts, qualitative and quantitative methods and other dimensions of relevance in a comprehensive approach towards a better understanding of the interrelation between travel behavior and health [20,21]. The PASTA project included, amongst other approaches, workshops and interviews with selected key stakeholders and a longitudinal population survey, which will be used for this study. The seven participating European CSCs (Antwerp, Barcelona, London, Örebro, Rome, Vienna, and Zurich) provided a good range in terms of size, urban environments, transport provision, modal split and ambition to increase levels of AM through cycling and walking [22,23].

### 2.2. Data Collection

For the interviews and workshops carried out in 2015, guidelines were developed at the beginning of the project to support a systematic and comparable approach in all PASTA cities. Fifteen to twenty key stakeholders were invited to a stakeholder workshop, and a sub-selection were then invited to in-depth interviews. To identify relevant stakeholders, a matrix served as guidance for the local CSC partners, combining topics (walking, cycling, health, transport, traffic safety, energy/climate, public transport, urban planning, stakeholder engagement/education/awareness raising) with types of stakeholders (political decision maker, public administration, business, association/NGO, and academia) [5]. In the introductory workshops, the stakeholders discussed challenges and barriers to AM promotion in each CSC [6]. Minutes were taken for the workshops and these were summarized into a standardized reporting template, resulting in 11–24 page reports per city. For the face-to-face interviews, the relevant sectors and players to be covered were defined (e.g., transport planning, urban planning, health, transport associations, etc.). An interview guide was available for the local CSC partners, containing a set of core questions (e.g., on framework conditions, planning processes, interventions to promote AM, etc.), which was complemented with questions pertaining to the local context, as needed [7]. Six to twelve interviews of 7 to 14 questions each were carried out per city. Each interview was transcribed and summarized in English into a standardized interview report template, resulting in 6–8 page reports.

Participants for the longitudinal survey were recruited opportunistically on a rolling basis [8]. A total of 10,691 participants entered the PASTA survey between November 2014 and November 2016, of which, 8567 completed a baseline questionnaire (BLQ) [22]. Participants provided detailed information on travel behavior and attitudes towards transport, amongst other subjects, as well as socio-demographic characteristics. The study also included regular short follow up questionnaires and a final questionnaire, but these were not used for the current analysis. Participants were required to be 18 years of age or older (16 years in Zurich), and had to give informed consent at registration. Data handling and ethical considerations regarding confidentiality and privacy of the information collected were reported in the study protocol [21].

A purposive literature search was carried out in December 2016 to ascertain whether an already existing policy scoring system could be used. Science Direct, PubMed and JSTOR were searched using the combination of “cycl*, walk*, policy, indicator, score, audit” in the title and abstract. In addition, a Google search with the same search terms was conducted to retrieve any additional grey literature.

### 2.3. Scoring Development

Based on the literature findings and on an assessment of available data collected through the PASTA project, a scoring framework of the walking and cycling policy friendliness was developed. To carry out the scoring in each CSC, a spreadsheet was developed, detailing the score items definition and description and indicating useful sections and questions to be consulted in the interview and workshop reports, respectively. Between March and August 2017, each of the score items was independently rated by one PASTA project expert and one or two local CSC experts, using score levels from zero to four, namely:

0—not existing, no evidence of recognition or reflection
1—existing but quite limited, low level of recognition or reflection
2—some reflection, existence and recognition; ok but not perfect, average
3—quite a lot existing, good reflection and recognition
4—very much existing, great reflection and recognition, we could not wish for much more.

The two independent scorings by the local and the project experts were recorded in a spreadsheet, along with the sources used and specific statements taken from the interview or workshop reports. If no statements were found for a specific item in the reports of a city, this was recorded as missing information, and if possible, knowledge of the local expert was used to derive a scoring of this item. Subsequently, the two scorings were compared and discussed in a phone conference. Disagreements were resolved by discussion. The basis for the final scoring of each item was also recorded in the merged spreadsheet. For one scoring item on which no interview question had been asked, data from the PASTA BLQ were used. As a proof of concept, the draft scoring system was initially applied to two cities (London and Vienna) to test the clarity of the explanations and feasibility of the scoring approach by two experts. Subsequently, amendments were made as necessary and the scoring was applied to the remaining cities. As Rome had applied a slightly different approach to the workshops and interviews, their data were used for the sensitivity analysis only.

Finally, the validity of the scoring was assessed with different approaches: interrater agreement (i.e., the extent to which the two raters assigned the same value for each item being rated) was evaluated for all cities using Cohen’s Kappa [24]. Correlations with the social norms towards cycling or walking were assessed, using the average of the following two questions from the PASTA BLQ: “People who are important to me think I should walk/cycle more” and “In my neighborhood, walking/cycling is well regarded” (each applying a five-point answering scale from “very much agree” to “very much disagree”). Furthermore, Pearson correlations were assessed (including pairwise complete observations), with the modal share of cycling or walking in each CSC, respectively, based on 2017 data from the European Platform on Mobility Management (EPOMM) Modal Split Tool (TEMS) [25], as reported in Mueller et al. [26]. In addition, for the cycling score, correlation with the length of the cycling network per 100,000 inhabitants was assessed, with data from OpenStreetMaps using labels of designated, non-shared cycling ways [27] as presented in Mueller et al. [26]. All analyses were carried out with the Statistical Package R [28].

## 3. Results

### 3.1. Literature Search and Scoring Approach

Several quantitative scoring systems on walking or cycling exist (e.g., Walk Score, based on walking routes to destinations such as grocery stores, schools, parks, restaurants, and retail [16] or an index assessing the readiness of cities to be bicycle-friendly based on socioeconomic and urban characteristics [29]). However, the literature search revealed only a few scoring systems with a qualitative approach, including aspects of policy supportiveness. Results included, for example, Bypad, a bicycle policy audit based on a guided self-assessment [19], and the former EU project PRESTO (Promoting cycling for everyone as daily transport mode [30]), however, these were developed as a series of policy guides rather than a scoring approach. Taking into account the goal of creating an evidence-based index of cycling/walking policy friendliness using qualitative data available from the interviews and workshops to complement the quantitative data from the PASTA survey, the most useful scoring system identified was the Copenhagenize index. This index is a city benchmarking system on cycling developed by an urban design consultancy in 2011, which is applied to many cities biannually, of which the top-ranked 100 cities’ scores are publicly available [31]. The 13 score parameters (as of 2017) could be grouped into three main areas: (1) usage (including mode share, gender split and mode share development); (2) facilities/infrastructure (including cycling facilities, cycling infrastructure, a bike sharing program and traffic calming measures); and (3) social and policy environment (including advocacy, bicycle culture, politics, social acceptance, perception of safety and urban planning). As the main goal of this study was to focus on the policy and social environment, the policy score was constructed from the six items of the third topical area “social and policy environment”. As the Copenhagenize methodology has not been published scientifically, only general information was available, which is summarized in Table 1 [32]. Based on this information, and taking into account the available data from the PASTA study, the score definitions to be used were developed for the cycling policy environment, and a corresponding, separate score was developed for the walking policy environment (see Supplementary Materials, Figures S1 and S2). As in the PASTA study no interview question had been asked on perceived traffic safety, we decided to use data on this topic from the PASTA BLQ, namely: “With your day-to-day travel needs in mind would you say that cycling “for travel” is safe (with regard to traffic)?”, with a 5-point answering scale from “very much agree” to “very much disagree”. A similar question was asked on “walking for travel”. In order to gain an insight into general traffic safety perceptions, rather than the views of those who have already become accustomed to the existing levels of (possibly even low levels of) traffic safety, the data of reported “never cyclists” (*n* = 1940) and “never walkers” (*n* = 1940), respectively, were used for the score on perceived traffic safety.

With six scoring items rated on a scale from zero (not existing, no evidence of recognition or reflection) to four (very much existing, great reflection and recognition, we could not wish for much more), the scoring ranged from 0 to a maximum of 24 points.

A spreadsheet was developed to carry out the scoring (see Supplementary Materials, Figure S2). For each scoring item, the specific questions to be consulted were specified (see Supplementary Materials, Figure S1), including the following interview questions:

(1) How pedestrian/cyclist friendly is the city? What are the greatest challenges? What has to be changed/improved?
(2) Which overall strategies exist to support AM in city x?
(3) What is the role of your institution, what AM measures/policies are you involved in?
(4) How was the health argument considered?
(5) Is there cooperation between health and transport/mobility sectors?
(6) What are the challenges supporting AM and implementing AM measures in city x?

From the workshop reports, the following questions were consulted for the items, as specified (see Supplementary Materials, Figure S1):

(1) Which framework conditions were advantageous in city x (for the implementation of AM measures)?
(2) What are the most important conditions that need to be in place for measures to be successful?
(3) What are the main barriers? What are the reasons for the fact that the measures suggested were not implemented so far? Is there a reason why they failed?

The perception-of-safety score item was constructed as the sum of percentages of PASTA baseline survey participants answers, as follows: “agree” plus (“very much agree” × 2) minus “disagree” minus (“very much disagree” × 2). Thus, a double weight was given to the “very much” (agree/disagree) categories, and no value was given to the neutral middle category. This resulted in a range of values from −53.3 (Antwerp) to −109.7 (London) for cycling and 30.2 (Barcelona) to 73.7 (London) for walking. Scores were assigned across the range of values, namely, from 0 (not existing, no evidence of recognition or reflection) for a value of ≥−125 for cycling and ≥70 for walking, up to 4 (very much existing, great reflection and recognition, we could not wish for much more) for a value of ≤−50 for cycling and <9 for walking, respectively (see Supplementary Materials, Tables S1 and S2).

### 3.2. Scoring across PASTA Cities

In Table 2, the results for each policy friendliness score item per city for walking and cycling are shown, respectively. Discussion of the results and resolution of any disagreements was feasible in all cases within a maximum of 75 min per city.

The results show that rating the policy friendliness for cycling and walking separately was appropriate, as different cities received the highest scores: the policy environment for cycling was rated as most supportive in Antwerp and Örebro, while in Zurich and Vienna it received the lowest scores. However, for walking, Zurich’s policy environment was rated the highest, followed by Örebro, while Antwerp’s was rated lowest. The picture across the different score items varied across cities, e.g., the item “advocacy” (i.e., the recognition and influence of the city’s cycling advocacy NGOs) received a high scoring in the relatively cycling policy friendly city of Antwerp, but a lower scoring in the similarly highly rated city of Örebro. The item “urban planning” (i.e., the emphasis the city’s planners place on bicycle or pedestrian infrastructure) was rated similarly for Antwerp and Barcelona, despite the latter’s much worse overall rating of cycling policy environment. This was also the case for the item “perceived traffic safety—walking” which received the same rating for Barcelona and Antwerp, despite Antwerp having a higher walking policy environment score. This seems to indicate that the overall scoring was not driven by a few scoring items but that each of them contributed (and in different ways across cities) to the overall final score.

### 3.3. Interrater Agreement and Validation

Overall, the interrater agreement of the five qualitative scores between the two experts was fair, both for cycling (ϰ = 0.211, *p* = 0.122) and for walking (ϰ = 0.211, *p* = 0.0648). These results did not change significantly when the scorings for Rome were included (cycling: ϰ = 0.228/*p* = 0.0684; walking: ϰ = 0.179/*p* = 0.119).

**Figure 1:**
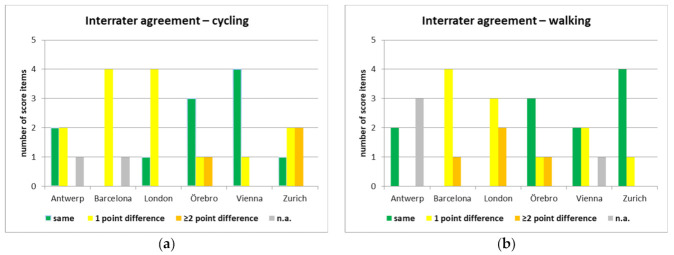
Interrater agreement per case study city, shown as number of five score items that were rated the same by both experts, with a one-point difference, or with a two-point or more difference on the rating scale from zero to four. (**a**) Interrater agreement for the policy friendliness score on cycling; (**b**) Interrater agreement for the policy friendliness score on walking.

As shown in Figure 1, the level of agreement also differed between the scoring on cycling and on walking for most cities. For example, we found a rather high level of agreement for London on the cycling policy environment versus a higher level of difference for walking, in addition to a high level of agreement for the scoring on Zurich for the walking policy environment but a higher level of difference on cycling.

**Figure 2:**
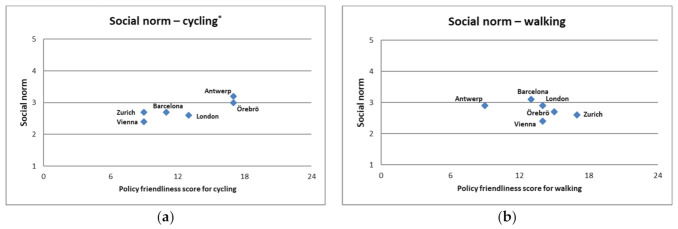
Correlation of the scoring of the policy friendliness score with the social norms, measured as average of two questions in the PASTA baseline questionnaire (“People who are important to me think I should walk/cycle more” and “In my neighborhood, walking/cycling is well regarded”, each applying a 5-point answering scale from “very much agree” to “very much disagree”). * = *p* < 0.05. (**a**) Correlation between social norm and policy friendliness score on cycling (r = 0.890, *p* = 0.008 *); (**b**) Correlation between social norm and the policy friendliness score on walking (r = −0.259, *p* = 0.575).

Comparing PASTA survey respondents’ own perception of social norms to the stakeholder-derived policy environment score, we found a relatively high correlation between the two for cycling (r = 0.89, *p* = 0.008), but a low and not statistically significant correlation for walking (r = −0.259, *p* = 0.575) (Figure 2, and Supplementary Materials, Table S3). The results did not differ significantly when the scorings for Rome were included (cycling: r = 0.891, *p* = 0.017; cycling: r = −0.460, *p* = 0.358).

**Figure 3:**
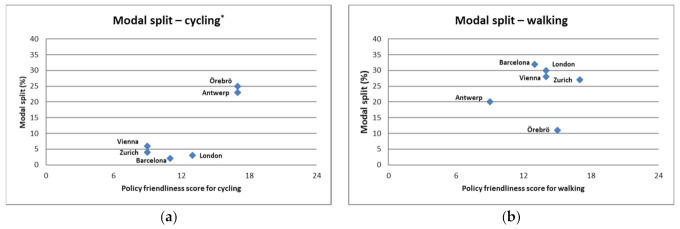
Correlation of the scoring of the policy friendliness score with the modal split of cycling or walking, measured as percentage of the total transport volume, * = *p* < 0.05. (**a**) Correlation between modal split and policy friendliness score on cycling (r = 0.89, *p* = 0.006 *); (**b**) Correlation between modal split and the policy friendliness score on walking (r = 0.333, *p* = 0.465).

Figure 3 shows a similar picture for the correlations with the modal split (Supplementary materials, Table S3): the correlation with the cycling policy environment was high (r = 0.89, *p* = 0.006), while it was statistically insignificant with the walking scoring (r = 0.333, *p* = 0.465). While for cycling the correlation was almost unchanged with the inclusion of Rome in the analysis (r = 0.88, *p* = 0.019), it was lower and still statistically insignificant for walking (r = 0.074, *p* = 0.890).

The policy friendliness score for cycling was also strongly correlated with the cycling network length per 100,000 inhabitants (Supplementary materials, Table S3), albeit with a correlation that was only borderline statistically significant (r = 0.751, *p* = 0.052), which remained largely unchanged when the scoring for Rome was included (r = 0.720, *p* = 0.103).

While Copenhagenize do not provide detailed scoring information per city, it can be noted that Antwerp—as one of the two most highly ranked PASTA CSCs for its cycling policy environment—has also been among the top-20 cities of Copenhagenize since 2013 [31]. London and Vienna have also featured on the Copenhagenize rankings for different years. As only cities with more than 600,000 inhabitants are rated, Örebro is not included in their ranking. In addition, results cannot be directly compared, as our approach focused solely on the social and policy environment aspects, while other dimensions are also included in the Copenhagenize ranking [32].

## 4. Discussion

The importance of providing a supportive policy environment for the promotion of walking and cycling has been underlined by international policy frameworks as part of a comprehensive approach to promoting sustainable and healthy urban transport [4,10,11] and to addressing climate change in urban settings [12,13,14]. Nevertheless, to our knowledge, this is the first science-driven approach (i.e., including assessments for reliability, interrater agreement, etc.) to scoring the supportiveness of the policy environment for walking and cycling.

In general, the results have proven the feasibility of such a scoring system, based on mostly qualitative data from semi-standardized stakeholder interviews and workshops. Our approach has a number of strengths: the available reports provided a wealth of information, and (with a few exceptions) also allowed the non-locally based expert to carry out a scoring of the items at hand. Carrying out two independent scorings added to the validity by combining on-the-ground knowledge of the local PASTA CSC expert with a more independent view by the general project expert, in addition, ensuring a more similar approach across all CSCs. Resolving any disagreements for a final scoring was feasible in a reasonable time frame. In addition, the approach allowed for validity testing, and results are encouraging, particularly for the policy area of cycling. Further confidence into the robustness of our approach stems from the results of the sensitivity analysis, which showed that including the scores of Rome (which had used a slightly different approach to the workshops and interviews) did not significantly change our results. 

On the other hand, a number of weaknesses must be noted: first of all, the PASTA stakeholder interviews and workshop were not constructed to serve this scoring. Their main purpose had been to collate and discuss policy approaches taken in the CSC and to provide context for the PASTA survey results [21,23]. While detailed guidance had been provided for the interviews and workshops [33,34,35], the approach allowed for a certain degree of flexibility across CSCs, which lead to some variance of questions asked in the different cities, as well as the level of detail in the available reports. This is apparent in the fair interrater agreement.

It was also noticeable that connotations of the term “active mobility”, which was often used in the interviews and workshops, differed across cities: overall, more information was available on cycling than on walking. This might explain the lower results found for the validity of the policy environment scoring for walking than for cycling. However, the difference in scoring results for walking versus cycling found in different cities is itself an interesting result. It confirms the assumption that cycling and walking are not necessarily given the same attention in local policy contexts. As space available for transport infrastructure is naturally limited in most cities, it is often difficult to allocate additional space to pedestrians, cyclists, and public transport at the same time. It is therefore likely that a “natural selection” will occur (explicitly or implicitly) in the policy process over time, which leads to focusing interventions and investments on either cycling or on public transport and walking (as walking is related to the use of public transport [36,37], given that typical walk trips occur to and from public transport stops). This selection process is likely to occur at least as long as taking away street space from motorized transport remains politically delicate in most cities as of yet. Thus, it can be expected that in cities where a high level of importance is given to public transport, the policy environment is also more mindful of the pedestrian needs, while cyclists might see less transport planning investments. Zurich and Barcelona are examples of such a policy environment: both have high levels of public transport use as well as levels of walking but comparatively lower importance is given to cycling [23], and thus lower levels of cycling use were observed [22,23]. On the other hand, for example, in Antwerp and Örebro, high levels of cycling were found [22] along with more cycling infrastructure thanks to a history of policy focus on cycling [23,26], while levels of walking (as well as public transport use) were comparatively lower. Thus, applying separate scorings for walking and cycling has proven to be justified. In future analyses, cycling and walking policy and infrastructure contexts should be treated separately, and not as a combination of “active travel” approaches.

For some items in some cities, no information was found in the reports; in particular, interviews had not always been carried out with the local advocacy groups. Finally, discussion to resolve disagreements also revealed a certain difference in the time orientation of the scoring: on the one hand, the interviews and workshops took place in 2015, while the scoring was carried out in 2017. While the non-local PASTA project expert relied mostly on the information provided in the reports, the local CSC expert was, at times, tempted to include more recent developments into his/her scoring. In some cases, conflicting statements on some scoring items by different stakeholders made it more challenging to come to an agreed final scoring. Finally, having two experts involved into the scoring (rather than a larger group of experts) could be seen as an additional weakness. On the other hand, increasing the number of experts involved would also significantly increase resource requirements, thus affecting the feasibility of such a scoring.

Overall, the results provide a number of lessons for the future: first of all, in future studies to confirm validity of our approach, a more systematic approach should be taken with regards to: (1) relevant stakeholders included across CSCs; (2) questions asked across cities; (3) separate questions asked on cycling and on walking; (4) specific questions asked on each item of the scoring. Reducing the scoring to four levels by removing the middle “neutral” category could also be considered to derive a clearer profile across cities. In addition, it could be interesting to compare the qualitative approach taken to analyze the information based on detailed accounts and statements with a more standardized, quantitative approach, e.g., using tools such as Nvivo [38]. This would, however, require recordings of each of the interviews, which was not the case in this project.

## 5. Conclusions

This unique study demonstrates the general feasibility of a qualitative approach to score the friendliness of the policy environment for walking and cycling. The PASTA project data offered a great opportunity to design and test such an approach. Replicating this approach in a more standardized way would pave the way towards developing a transparent, evidence-based system to benchmark cities, while informing policymaking and tracking progress over time in a policy area of increasing importance. In addition, capturing the wealth of qualitative information into a quantitative score would also allow for the inclusion of such information into statistical analyses of travel behavior. This would add an important aspect of the policy and cultural context to analyses of quantitative data, for example, in multivariate models assessing determinants of AM across cities, or when comparing prevalence and determinants of cycling or walking accidents across cities. Adding this element of policy context, which is not usually captured in travel behavior analyses, would increase data richness and the information value of such multivariate data analyses. Unfortunately, most of the PASTA BLQ quantitative data analyses had already been completed by the time we had finalized this feasibility study. In future analyses, the policy score could be included to test its explanatory value.

## Figures and Tables

**Table 1:** Indicator score items with summary definitions of Copenhagenize, PASTA score definitions and description (scored from level zero—not existing, no evidence of recognition or reflection to level four—very much existing, great reflection and recognition, we could not wish for much more).

Indicator Score Items and Sources of Data Used	Summary of Copenhagenize Definition *	PASTA Cycling Policy Friendliness Score Definition and Description	PASTA Walking Policy Friendliness Score Definition and Description
**Social Environment**			
(1) Culture °^1^	How present are bicycles in the urban landscape: only a few sporty cyclists to mainstream acceptance among regular citizens	Has the bicycle reestablished itself as a mode of transport among regular citizens or only sub-cultures? Description: No cyclists in the urban landscape to mainstream acceptance of cyclists	Has walking reestablished itself as a mode of transport among regular citizens or only sub-cultures? Description: No pedestrians in the urban landscape to mainstream acceptance of pedestrians
(2) Social acceptance °^2^	Level of social acceptance of urban cyclists as a respected, accepted and normal form of transport	How do drivers and the community at large regard urban cyclists? Description: No social acceptance to widespread social acceptance	How do drivers and the community at large regard urban pedestrians? Description: No social acceptance to widespread social acceptance
(3) Perception of safety °^3^	Is the perception of safety of the cyclists in the city, reflected in helmet-wearing rates, positive or are cyclists riding scared due to helmet promotion and scare campaigns?	With your day-to-day travel needs in mind, would you say that cycling “for travel” is safe (with regards to traffic)? Description: 5-pt scale: “very much disagree” to “very much agree”	With your day-to-day travel needs in mind, would you say that walking “for travel” is safe (with regards to traffic)? Description: 5-pt scale: “very much disagree” to “very much agree”
**Policy Environment**			
(4) Advocacy °^2^	Level of activity of local advocacy to encourage citizens to cycle, e.g., through public campaigns, and contribution to local policy	How is the city’s cycling advocacy NGO(s) regarded and what level of influence does it have? Description: No organized advocacy to strong advocacy with political influence	How is the city’s pedestrian advocacy NGO(s) regarded and what level of influence does it have? Description: No organized advocacy to strong advocacy with political influence
(5) Politics °^1^	Level of support by politicians for quality bike infrastructure, streamlined planning processes and use of bikes by politicians	Political climate regarding urban cycling Description: non-existent on a political level to active and passionate political involvement	Political climate regarding urban walking Description: non-existent on a political level to active and passionate political involvement
(6) Urban Planning °^2^	Level of development of network of infrastructure, testing of innovative ideas and availability of dedicated planning office for bicycle infrastructure	Emphasis the city’s planners place on bicycle infrastructure Description: car-centric urban planners to planners who think in a bicycle first manner	Emphasis the city’s planners place on pedestrian infrastructure Description: car-centric urban planners to planners who think in a pedestrian first manner

* as of 2017 [32]. ° Sources of data used (for more details, see Supplementary Materials, Figures S1 and S2): ^1^ Stakeholder interview reports and local expert knowledge. ^2^ Stakeholder interview reports, Workshop reports and local expert knowledge. ^3^ Physical Activity through Sustainable Transport Approaches (PASTA) Baseline questionnaire.

**Table 2:** Results of the policy friendliness scoring for cycling and for walking, per city, as rated by two independent PASTA experts in 2017, and based on PASTA baseline survey data for the scoring item “perceived traffic safety”. Each item was scored on a scale of 0 (not existing, no evidence of recognition or reflection) to 4 (very much existing, great reflection and recognition, we could not wish for much more).

	Policy Friendliness Score for Cycling							Policy Friendliness Score for Walking						
	Culture	Social Acceptance	Perceived Traffic Safety *	Advocacy	Politics	Urban Planning	Total	Culture	Social Acceptance	Perceived Traffic Safety *	Advocacy	Politics	Urban Planning	Total
Antwerp	3	3	4	3	2	2	17	1	2	2	1	1	2	9
Barcelona	1	2	2	3	1	2	11	3	3	2	1	1	3	13
London	1	2	1	3	3	3	13	2	2	4	2	2	2	14
Örebro	3	3	3	1	4	3	17	2	4	3	1	2	3	15
Vienna	1	1	1	2	2	2	9	3	2	2	2	2	3	14
Zurich	2	1	2	2	1	1	9	3	3	2	2	3	3	16
*Rome ^1^*	*1*	*1*	*2*	*2*	*2*	*1*	*9*	*2*	*1*	*0*	*2*	*2*	*1*	*8*

* Scoring based on PASTA baseline questionnaire question: “With your day-to-day travel needs in mind would you say that cycling “for travel” (or: walking “for travel”, respectively) is safe (with regards to traffic)?”, with a five-point answering scale from “very much agree” to “very much disagree”. *^1^* Due to a slightly different approach taken to the workshops and interviews, data were used for sensitivity analysis only. Italics: data was not used in the study.

## Data Availability

The data presented in this study are available in the Supplementary Materials.

## Supplementary Materials

The following are available online at https://www.mdpi.com/1660-4601/18/3/986/s1: Figure S1: Explanations on filling in the policy scoring sheet, Figure S2: Example of empty scoring sheets for cycling and for walking, Table S1: Results on perceived traffic safety scoring per city for cycling, Table S2: Results on perceived traffic safety scoring per city for walking, Table S3: Policy friendliness scoring for cycling and for walking and data used for validity testing, including modal splits for cycling and walking, cycling network length in km/100,000 inhabitants and social norm for cycling and walking, respectively.

## References

[1] 2018 Physical Activity Guidelines Advisory Committee (2018). 2018 Physical Activity Guidelines Advisory Committee Scientific Report.

[2] Sallis J.F., Bull F., Guthold R., Heath G.W., Inoue S., Kelly P., Oyeyemi A.L., Perez L.G., Richards J., Hallal P.C. (2016). Progress in Physical Activity over the Olympic Quadrennium. Lancet.

[3] Guthold R., Stevens G.A., Riley L.M., Bull F.C. (2018). Worldwide Trends in Insufficient Physical Activity from 2001 to 2016: A Pooled Analysis of 358 Population-Based Surveys with 19 Million Participants. Lancet Glob. Health.

[4] World Health Organization (2018). WHO Global Action Plan on Physical Activity 2018−2030: More Active People for a Healthier World.

[5] World Health Organization (2013). Global Action Plan for the Prevention and Control of NCDs 2013-2020.

[6] Kelly P., Kahlmeier S., Gotschi T., Orsini N., Richards J., Roberts N., Scarborough P., Foster C. (2014). Systematic Review and Meta-Analysis of Reduction in All-Cause Mortality from Walking and Cycling and Shape of Dose Response Relationship. Int. J. Behav. Nutr. Phys. Act..

[7] Gotschi T., Garrard J., Giles-Corti B. (2016). Cycling as a Part of Daily Life: A Review of Health Perspectives. Transp. Rev..

[8] WHO Regional Office for Europe (2015). WHO European Region Physical Activity Strategy 2016–2025.

[9] Edwards P., Tsouros A. (2006). Promoting Physical Activity and Active Living in Urban Environments.

[10] ISPAH International Society on Physical Activity and Public Health (2016). Bangkok Declaration on Physical Activity for Global Health and Sustainable Development.

[11] United Nations Economic Commission for Europe, WHO Regional Office for Europe (2014). Paris Declaration of the Fourth High Level Meeting on Transport, Environment and Health: City in Motion, People First.

[12] Woodcock J., Edwards P., Tonne C., Armstrong B.G., Ashiru O., Banister D., Beevers S., Chalabi Z., Chowdhury Z., Cohen A. (2009). Public Health Benefits of Strategies to Reduce Greenhouse-Gas Emissions: Urban Land Transport. Lancet.

[13] Sallis J.F., Spoon C., Cavill N., Engelberg J.K., Gebel K., Parker M., Thornton C.M., Lou D., Wilson A.L., Cutter C.L. (2015). Co-Benefits of Designing Communities for Active Living: An Exploration of Literature. Int. J. Behav. Nutr. Phys. Act..

[14] WHO Regional Office for Europe (2017). Towards More Physical Activity in Cities: Transforming Public Spaces to Promote Physical Activity-a Key Contributor to Achieving the Sustainable Development Goals in Europe.

[15] Bull F., Milton K., Kahlmeier S. (2014). National Policy on Physical Activity: The Development of a Policy Audit Tool (PAT). J. Phys. Act. Health.

[16] About Walk Score. https://www.walkscore.com/about.shtml.

[17] Winters M., Brauer M., Setton E.M., Teschke K. (2013). Mapping Bikeability: A Spatial Tool to Support Sustainable Travel. Environ. Plan. B Plan. Des..

[18] Lovelace R., Goodman A., Aldred R., Berkoff N., Abbas A., Woodcock J. (2017). The Propensity to Cycle Tool: An Open Source Online System for Sustainable Transport Planning. JTLU.

[19] Bypad. https://www.bypad.org/.

[20] Dons E., Götschi T., Nieuwenhuijsen M., de Nazelle A., Anaya E., Avila-Palencia I., Brand C., Cole-Hunter T., Gaupp-Berghausen M., Kahlmeier S. (2015). Physical Activity through Sustainable Transport Approaches (PASTA): Protocol for a Multi-Centre, Longitudinal Study. BMC Public Health.

[21] Gerike R., de Nazelle A., Nieuwenhuijsen M., Panis L.I., Anaya E., Avila-Palencia I., Boschetti F., Brand C., Cole-Hunter T., Dons E. (2016). Physical Activity through Sustainable Transport Approaches (PASTA): A Study Protocol for a Multicentre Project. BMJ Open.

[22] Raser E., Gaupp-Berghausen M., Dons E., Anaya-Boig E., Avila-Palencia I., Brand C., Castro A., Clark A., Eriksson U., Götschi T. (2018). European Cyclists’ Travel Behavior: Differences and Similarities between Seven European (PASTA) Cities. J. Transp. Health.

[23] Wegener S., Raser E., Gaupp-Berghausen M., Anaya E., Nazelle A.D., Eriksson U., Gerike R., Horvath I., Iacorossi F., Panis L. Active Mobility–the New Health Trend in Smart Cities, or Even More?. https://repository.corp.at/348/.

[24] Gwet K.L. (2014). Handbook of Inter-Rater Reliability, 4th Edition: The Definitive Guide to Measuring The Extent of Agreement Among Raters.

[25] TEMS-The EPOMM Modal Split Tool. http://www.epomm.eu/tems/about_tems.phtml.

[26] Mueller N., Rojas-Rueda D., Salmon M., Martinez D., Ambros A., Brand C., de Nazelle A., Dons E., Gaupp-Berghausen M., Gerike R. (2018). Health Impact Assessment of Cycling Network Expansions in European Cities. Prev. Med..

[27] OpenStreetMap Contributors Planet Dump. https://planet.openstreetmap.org/.

[28] R: The R Project for Statistical Computing. https://www.r-project.org/.

[29] Zayed M.A. (2016). Towards an Index of City Readiness for Cycling. Int. J. Transp. Sci. Technol..

[30] Promoting Cycling for Everyone as Daily Transport Mode-Intelligent Energy Europe-European Commission. /energy/intelligent/projects/en/projects/presto.

[31] (2019). Copenhagenize Index-Copenhagenize. https://copenhagenizeindex.eu/.

[32] Copenhagenize-Our Methodology. https://copenhagenizeindex.eu/about/methodology.

[33] Wegener S., Uhlmann T. (2014). Carsten Rothballer Guideline for the Interviews in the Case Study Cities (CSC). Physical Activity through Sustainable Transport Approaches (PASTA) Work Package 2: Assessment of AM Initiatives and Framework Factors in Case Study Cities.

[34] Wegener S., Uhlmann T. (2014). Carsten Rothballer Guideline for the Workshop in the Case Study Cities (CSC). Physical Activity through Sustainable Transport Approaches (PASTA). Work Package 2: Assessment of AM Initiatives and Framework Factors in Case Study Cities.

[35] Wegener S., Uhlmann T. (2014). Carsten Rothballer Guideline of the Selection of Stakeholders. Physical Activity through Sustainable Transport Approaches (PASTA) Work Package 2: Assessment of AM Initiatives and Framework Factors in Case Study Cities.

[36] Sallis J.F., Cerin E., Conway T.L., Adams M.A., Frank L.D., Pratt M., Salvo D., Schipperijn J., Smith G., Cain K.L. (2016). Physical Activity in Relation to Urban Environments in 14 Cities Worldwide: A Cross-Sectional Study. Lancet.

[37] Chaix B., Benmarhnia T., Kestens Y., Brondeel R., Perchoux C., Gerber P., Duncan D.T. (2019). Combining Sensor Tracking with a GPS-Based Mobility Survey to Better Measure Physical Activity in Trips: Public Transport Generates Walking. Int. J. Behav. Nutr. Phys. Act..

[38] Qualitative Data Analysis Software | NVivo. https://www.qsrinternational.com/nvivo-qualitative-data-analysis-software/home.

