# Supplementary material for: Assessing the Policy Environment for Active Mobility in Cities—Development and Feasibility of the PASTA Cycling and Walking Policy Environment Score
Source: Int J Environ Res Public Health. 2021 Jan 22;18(3):986. doi: 10.3390/ijerph18030986 (PMC7908172; doi:10.3390/ijerph18030986)
Supplement: Supplementary file 1 [file ijerph-18-00986-s001.pdf]

Assessing the policy environment for cycling and walking promotion in cities – development and feasibility of the PASTA cycling & walking policy environment score

Figure S1: Explanations on filling in the policy scoring sheet

**Policy score**				
**Additional explanations**				
**1. Approach to use for the scoring**				
1. An expert from the local PASTA team and the PASTA policy-score expert do an independent scoring of each item for walking and cycling separately. They both record sources of information and arguments for their scoring.				
	As data sources, mostly the interviews and workshop reports should be used. See further indications on interview questions and workshop sections to be used below. The indicared sections should be			
	scanned and useful sections pasted or summarized in the cell "explanation why score was given".			
	The local expert is invited to add additional perspectives and elements, if relevant (e.g. from media reports on cycling/walking, political discussions etc.).			
	No additional literature analysis is required.			
	If uncertain on a scoring (e.g. on advocacy, culture, acceptance), you may also compare the same item for the other mode (walking or cycling) - should the score be higher/lower for cycling/walking?			
2. The two scorings are merged by the PASTA expert into one table per city and shared with the local expert from the PASTA team.				
3. Disagreements are resolved through discussion in a phone meeting. Arguments for the agreed score are documented.				
**2. Score items**	**Definition***	**Score description***	**Additional explanations**	**Useful sources and specific questions°**
**Social environment**				Please scan the following sections for useful information:
**Bicycle / Pedestrian Culture**	Has the bicycle / walking reestablished itself as mode of transport among regular citizens or only sub-cultures?	no pedestrians / cyclists on the urban landscape / only hiking to mainstream acceptance	Here the actual practice of walking / cycling should be taken into account rather than the social perception on those modes (rate those under "social acceptance") below.	**interview questions on:**<br>1) walking/cycling-friendlines: "How pedestrian and cyclist friendly is the city? What are greatest challenges? What has to be changed / improved?"<br>2) current AM measures: "Which overall strategies exist to support AM in x?"
**Social Acceptance**	How do drivers and the community at large regard urban cyclists / pedestrians?	no social acceptance to widespread social acceptance.	How are walkers and cyclists regarded? This refers to the image, as reflected in media reports (esp. for cyclists - mostly positive or negative press reports?) as well as in statements by politicians and stakeholders (interviews, workshops). For walking being regarded as a "normal behaviour" not warranting media reports (=high score) or not being accepted as a mode of transport (= low score 0-1) could apply.	**Interview questions on:**<br>1) framework conditions: "Role of the institution, AM measures/policies they are involved in, how was health argument considered? "<br>2) walking/cycling-friendliness: "How pedestrian and cyclist friendly is the city? What are greatest challenges? What has to be changed / improved?"<br>3) barriers: "What are the challenges supporting AM and implementing AM measures in x?"<br>**Workshop section on:**<br>4) Framework conditions: "Which framework conditions were advantageous in city x (for the implementation of AM measures)?"<br>5) Success factors: "what are the most important conditions which need to be in place for measures to be successful"<br>6) Barriers: "What are the main barriers? What are the reasons for the fact that the measures suggested were not implemented so far? Is there a reason why they failed?"
**Perception of Safety**	With your day-to-day travel needs in mind, would you say that cycling / walking "for travel" is safe (with regards to traffic)?	(data: 5-pt scale - very much disagree - very much agree)	Score will be constructed using PASTA BLQ data, do not enter a score yourself.<br>For later validation, please also list any statemens made regarding traffic safety (perceived or real) of pedestrians/ cyclists in the respective city (e.g. in interview qu's on barriers, walking-/cycling-friendliness) (e.g. search for "accid", "injur", "safe").<br>Don't list general statements like "safety is important for cycling" but things like "there are many accidents in city x so cyclists feel unsafe").	search interviews for key words, see left

Figure S1 – continued

**Policy environment**				
**Advocacy**	How is the city's advocacy NGO(s) regarded and what level of influence does it have?	No organized advocacy to strong advocacy with political influence	Both the existence of an advocacy group as well as their actual activities should be taken into account. E.g. in some cases no specific adovcacy group may exist for walking (low score 0-1), or a general advocacy for sustainability may be very active on walking (high score 2-3). Strong specific advocacy group with political influence = score 4.<br>Accordingly, no advocacy because the topic is already well established would also be a low score.	**Interview with one of these groups, if existing**<br>**Workshop report on:**<br>1) framework conditions: "Which framework conditions were advantageous in city x (for the implementation of AM measures)? "<br>2) success factors: "what are the most important conditions which need to be in place for measures to be successful?"<br>3) barriers: "What are the main barriers? What are the reasons for the fact that the measures suggested were not implemented so far? Is there a reason why they failed?"<br>**or knowledge of the local team**
**Politics**	Political climate regarding urban cycling / walking	being non-existent on a political level to active and passionate political involvement	Here the political practice and processes should be the main basis for decision, e.g. the existence of political leadership, reflection in political discussions and recognition of cycling/walking by politicians and policy makers. Reflection in policy documents, allocation of dedicated funding can also be taken into account but this is mostly about the "climate".	**Interview questions on:**<br>1) current AM measures: "Which overall strategies exist to support AM in x? "<br>2) collaboration: "Is there cooperation between health and transport/mobility sector?"<br>3) walking/cycling-friendliness: "How pedestrian and cyclist friendly is the city? What are greatest challenges? What has to be changed / improved?"<br>4) barriers:"What are the challenges supporting AM and implementing AM measures in x? "
**Urban Planning**	How much emphasis do the city's planners place on pedestrian/cycling infrastructure	car-centric urban planners to planners who think (bicycle - and) pedestrian - first	This should mostly reflect current practice in transport planning but can also contain an element of existence of policy documents meant to influence planning practice.	**Interview questions on:**<br>1) current AM measures: "Which overall strategies exist to support AM in x? "<br>2) walking/cycling-friendliness:""How pedestrian and cyclist friendly is the city? What are greatest challenges? What has to be changed / improved?"<br>3) barriers:"What are the challenges supporting AM and implementing AM measures in x?<br>4) framework conditions:"Role of the institution, AM measures/policies they are involved in, how was health argument considered? "<br>**Workshop section on:**<br>5) framework conditions: "Which framework conditions were advantageous in city x (for the implementation of AM measures)? "<br>6) success factors: "what are the most important conditions which need to be in place for measures to be successful?"<br>7) barriers: "What are the main barriers? What are the reasons for the fact that the measures suggested were not implemented so far? Is there a reason why they failed?"

Figure S1 – continued 2

Figure S1 – continued

3. Score levels (0-4)	Additional explanations
0	not existing, no evidence of recogition or reflection
1	existing but quite limited, low level of recognition or reflection
2	some reflection, existence and recognition - ok but not perfect, average
3	quite a lot existing, good reflection and recognition
4	very much existing, great reflection and recogntion, we could not wish for much more (we are probably not that far from Copenhagen or Amsterdam)

* based on Copenhagenize, except for perception of safety

° Note: Questions were not asked the same way in all cities and sometimes questions were amended or dropped - pls scan the reports for sections that address such themes.

4. Further specifications regarding useful questons in the interviews / workshops:

**Theme**	**Interview question(s)**
walking/cycling-friendlin	How pedestrian and cyclist friendly is the city? What are greatest challenges? What has to be changed / improved?
current AM measure	Which overall strategies exist to support AM in x?
barriers	What are the challenges supporting AM and implementing AM measures in x?
collaboration	Is there cooperation between health and transport/mobility sector?
	In which way cooperation takes place?
framework conditions	Role of the institution, AM measures/policies they are involved in, how was health argument considered?
	**Workshop questions/sections**
framework conditions	Which framework conditions were advantageous in city x (for the implementation of AM measures)?
success factors	what are the most important conditions which need to be in place for measures to be successful?
barriers	What are the main barriers? What are the reasons for the fact that the measures suggested were not implemented so far?
	Is there a reason why they failed?

CITY x											
Cycling				PLEASE FILL IN				PLEASE FILL IN	PLEASE FILL IN		
**Item**	**Definition**	**Score description (0-4)**	**Possible PASTA source(s) Please see sheet "explanations"**	**Score CITY x local expert (0-4)**	**Score CITY x PASTA expert (0-4)**	**Agreed score**	**Arguments for agreement**	**Source used local expert**	**Explanations why this score was given: CITY x local expert**	**Source used PASTA expert**	**Explanations why this score was given: PASTA expert**
**Social environment**											
**1 Bicycle Culture**	**Has the bicycle reestablished itself as a mode of transport among regular citizens or only sub-cultures?**	*no bicycles on the urban landscape / only sporty cyclists to mainstream acceptance*	*Interviews*								
**2 Social Acceptance**	**How do drivers and the community at large regard urban cyclists?**	*no social acceptance to widespread social acceptance.*	*Interviews / workshops / local partners knowledge*								
**3 Perception of Safety**	*Score will be constructed using PASTA BLQ data for your city*	*In addition, to validate the quantitative score please list any statements on cycling traffic safety from the interview/workshop reports*		x	x	x	x		Relevant statements found:		Relevant statements found:
**Policy environment**											
**4 Advocacy**	**How is the city's advocacy NGO(s) regarded and what level of influence does it have?**	*No organized advocacy to strong advocacy with political influence*	*interviews/workshops - partners knowledge*								
**5 Politics**	**Political climate regarding urban cycling**	*being non-existent on a political level to active and passionate political involvement*	*Interviews / workshops*								
**6 Urban Planning**	**How much emphasis do the city's planners place on bicycle infrastructure**	*car-centric urban planners to planners who think bicycle (- and pedestrian) - first*	*Interviews*								
			Total	0	0	0					
				(0-24)							

CITY x											
Walking											
				PLEASE FILL IN				PLEASE FILL IN	PLEASE FILL IN		
**Item**	**Definition**	**Score description (0-4)**	**Possible PASTA source(s)**	**Score CITY x local expert (0-4)**	**Score CITY x PASTA expert (0-4)**	**Agreed score**	**Arguments for agreement**	**Source used**	**Explanations why this score was given**	**Source used PASTA expert**	**Explanations why this score was given - PASTA expert**
**Social environment**											
**1 Walking Culture**	**Has walking (for transport) reestablished itself as a mode of transport among regular citizens or only sub-cultures?**	*no pedestrians on the urban landscape / only hiking to mainstream acceptance*	*Interviews*								
**2 Social Acceptance**	**How do drivers and the community at large regard urban pedestrians?**	*no social acceptance to widespread social acceptance.*	*Interviews / workshops / local partners knowledge*								
**3 Perception of Safety**	*Score will be constructed using PASTA BLQ data for your city*	*In addition, to validate the quantitative score please list any statements on walking traffic safety from the interview/workshop reports*	*Workshop, interviews*	x	x	x	x		Relevant statements found:		Relevant statements found:
**Policy environment**											
**4 Advocacy**	**How is the city's advocacy NGO(s) regarded and what level of influence does it have?**	*No organized advocacy to strong advocacy with political influence*	*interviews/workshops - partners knowledge*								
**5 Politics**	**Political climate regarding urban pedestrians**	*being non-existent on a political level to active and passionate political involvement*	*Interviews / workshops*								
**6 Urban Planning**	**How much emphasis do the city's planners place on pedestrian infrastructure**	*car-centric urban planners to planners who think (bicycle - and) pedestrian - first*	*Interviews*								
			Total	0	0	0					
				(0-24)							

Figure S2: Example of empty scoring sheets for cycling and for walking

Table S1: Results on perceived traffic safety scoring per city for cycling

	N	Antwerp	Barcelona	London	Örebro	Vienna	Zurich	*Rome*[3]
Very much agree	42	4.7	1.6	0.2	2.4	1.6	1.4	*4.5*
Agree	148	18.6	4.6	5.2	9.5	6.5	6.5	*12.1*
Neither agree/disagree	360	16.3	18.6	20.6	35.7	15.2	14.8	*17.0*
Disagree	760	39.5	47.9	32.5	23.8	41.3	48.1	*33.9*
Very much disagree	630	20.9	27.3	41.4	28.6	35.3	29.2	*32.5*
Total	1940	100.0	100.0	100.0	100.0	100.0	100.0	100.0
Score value[1]		-53.5	-94.6	-109.7	-66.7	-102.2	-97.2	*-77.7*
**Scoring**[2]		**4**	**2**	**1**	**3**	**1**	**2**	***1***

[1], Based on question "With your day-to-day travel needs in mind would you say that cycling 'for travel' is safe (with regards to traffic)." (category: never-cyclists). Score value = sum of percentages: "agree" plus "very much agree"x 2 minus "disagree" minus "very much disagree"x 2.

[2] Scoring scale: ≥ -125 = 0, -100 - -124.9 = 1, -75 - -99.9 = 2, - 50 - -74.9 = 3, ≤ -50 = 4

[3] Due to a slightly different approach taken to the workshops and interviews, data was used for sensitivity analysis only

Table S2: Results on perceived traffic safety scoring per city for walking

	N	Antwerp	Barcelona	London	Örebro	Vienna	Zurich	*Rome*[3]
Very much agree	283	14.6%	9.3%	16.6%	11.4%	15.7%	17.9%	*9.5%*
Agree	850	43.8%	46.5%	53.5%	46.9%	39.5%	33.2%	*33.3%*
Neither agree/disagree	437	22.5%	18.6%	19.4%	26.8%	17.0%	27.7%	*28.6%*
Disagree	249	12.8%	16.3%	7.8%	12.2%	17.4%	12.0%	*11.9%*
Very much disagree	121	6.2%	9.3%	2.6%	2.7%	10.4%	9.2%	*16.7%*
Total	1940	100.0%	100.0%	100.0%	100.0%	100.0%	100.0%	*100.0%*
Score value*		47.7	30.2	73.7	52.1	32.7	38.6	*7.1*
**Scoring**		**2**	**2**	**4**	**3**	**2**	**2**	***0***

[1], Based on question "With your day-to-day travel needs in mind would you say that walking 'for travel' is safe (with regards to traffic)." (category: never-walkers). Score value = sum of percentages: "agree" plus "very much agree"x 2 minus "disagree" minus "very much disagree"x 2.

[2] Scoring scale: <9 = 0, 28.9 – 9 = 1, 48.9 – 29 = 2, 69.9 – 49 = 3, ≥ 70 = 4

[3] Due to a slightly different approach taken to the workshops and interviews, data was used for sensitivity analysis only

Table S3: Policy friendliness scoring for cycling and for walking and data used for validity testing, including modal splits for cycling and walking, cycling network length in km/100'000 inhabitants and social norm for cycling and walking, respectively.

City	Score cycling	Score walking	Modal split[1] Cycling, %	Modal split[1] walking, %	Cycle network[2] km / 100k	Social norm cycling[3]	Social norm walking[3]
**Antwerp**	17	9	23	20	91	3.2	2.9
**Barcelona**	11	13	2	32	10	2.7	3.1
**London**	13	14	3	30	11	2.6	2.9
**Orebro**	17	15	25	11	256	3	2.7
**Vienna**	9	14	6	28	40	2.4	2.4
**Zurich**	9	16	4	27	29	2.7	2.6
Rome[4]	*9*	*8*	*1*	*16*	*4*	*2.6*	*2.7*

[1] Measured as percentage of the total transport volume, based on 2017 data from the European Platform on Mobility Management (EPOMM) Modal Split Tool (TEMS) [1] as reported in Mueller et al. [2].

[2] data from OpenStreetMaps using labels of designated, non-shared cycling ways [3] as presented in Mueller et al. [2]

[3] Social norm measured as average of two questions in the PASTA baseline questionnaire: "People who are important to me think I should walk/cycle more" and "In my neighbourhood, walking/cycling is well regarded", each applying a a 5-point answering scale from "very much agree" to "very much disagree".

[4] Due to a slightly different approach taken to the workshops and interviews, data was used for sensitivity analysis only.

References

1. TEMS - The EPOMM Modal Split Tool Available online: http://www.epomm.eu/tems/about_tems.phtml (accessed on Nov 5, 2020).
2. Mueller, N.; Rojas-Rueda, D.; Salmon, M.; Martinez, D.; Ambros, A.; Brand, C.; de Nazelle, A.; Dons, E.; Gaupp-Berghausen, M.; Gerike, R.; et al. Health impact assessment of cycling network expansions in European cities. *Preventive Medicine* **2018**, *109*, 62–70, doi:10.1016/j.ypmed.2017.12.011.
3. OpenStreetMap contributors Planet Dump Available online: https://planet.openstreetmap.org/ (accessed on Nov 5, 2020).
